# An advance in transfer line chilldown heat transfer of cryogenic propellants in microgravity using microfilm coating for enabling deep space exploration

**DOI:** 10.1038/s41526-021-00149-5

**Published:** 2021-06-08

**Authors:** J. N. Chung, Jun Dong, Hao Wang, S. R. Darr, J. W. Hartwig

**Affiliations:** 1 Space Cryogenics Thermal Energy Management Laboratory, Department of Mechanical and Aerospace Engineering, University of Florida, Gainesville, FL USA; 2 NASA Glenn Research Center, Cleveland, OH USA

**Keywords:** Aerospace engineering, Environmental chemistry

## Abstract

The extension of human space exploration from a low earth orbit to a high earth orbit, then to Moon, Mars, and possibly asteroids is NASA’s biggest challenge for the new millennium. Integral to this mission is the effective, sufficient, and reliable supply of cryogenic propellant fluids. Therefore, highly energy-efficient thermal-fluid management breakthrough concepts to conserve and minimize the cryogen consumption have become the focus of research and development, especially for the deep space mission to mars. Here we introduce such a concept and demonstrate its feasibility in parabolic flights under a simulated space microgravity condition. We show that by coating the inner surface of a cryogenic propellant transfer pipe with low-thermal conductivity microfilms, the quenching efficiency can be increased up to 176% over that of the traditional bare-surface pipe for the thermal management process of chilling down the transfer pipe. To put this into proper perspective, the much higher efficiency translates into a 65% savings in propellant consumption.

## Introduction

The currently planned lower-earth-orbiting propellant space depots and the human orbital transfer spacecraft to the moon and Mars will need to depend on the high thrust and high efficiency of liquid cryogenic chemical propulsion or nuclear thermal propulsion^1–4^. A highly efficient transfer process for in-space tank-to-tank propellant transfer (propellant depot to orbital transfer spacecraft) of cryogenic propellants is an enabling technology for future Crewed Mars Surface Mission^1^. The transfer of cryogenic propellants in space, however, has yet to be accomplished, mainly owing to the unavailability of cryogenic two-phase quenching heat transfer data of a transfer line in reduced gravity and microgravity^4^. When a cryogenic propellant such as liquid hydrogen (LH_2_) or liquid oxygen (LOX) is first introduced into a warm pipe from a depot supply tank to an engine or a receiver storage tank, a chilldown process follows where the liquid propellant boils into vapor until the transfer pipe and the receiving tank are quenched down to the liquid propellant temperature. After this transient “chilldown” procedure, single-phase liquid propellant can be transferred to the destination for designated uses such as the rocket engine fuel for combustion. The spent propellant during chilldown is a two-phase mixture of vapor and liquid that cannot be used for any purpose and therefore must be vented overboard. Since the current chilldown technology can only manage to offer relatively very low thermal energy efficiencies^5^ and further that it has never been developed under space microgravity conditions, a new breakthrough technology advancement that significantly raises these efficiencies, and is also verified under space conditions is needed for enabling deep space missions.

Experimental studies of quenching (chilldown) of a cryogenic transfer pipe in terrestrial gravity were attempted initially without much success in the 1960s^6,7^. However, only within the last decade has the terrestrial cryogenic chilldown research been renewed with some success^8–10^ and recently a complete LN_2_ chilldown dataset with measured heat transfer coefficients for all quenching regimes over a large range of conditions was reported^11–15^.

Because of the large differences in densities between the vapor and liquid, both flow patterns and heat transfer characteristics during quenching on earth are quite different from those in microgravity^10,16,17^. Microgravity chilldown (quenching) experiments using refrigerants R-113 and FC-72 fluids have been reported^18–20^. There has been very little data reported for the quenching of cryogenic fluids in microgravity due to experimental difficulties. In 1990s, Antar and Collins^21,22^ reported qualitative results due to many equipment deficiencies for a limited range of conditions from a single reduced gravity LN2 chilldown experiment using a stainless steel bare surface test tube. Almost 20 years later, the group at the University of Florida that reported the complete terrestrial LN_2_ chilldown experimental dataset mentioned above^8,11–15^ also performed parabolic flight traditional chilldown experiments onboard a C9 aircraft and reported a wide ranging dataset of microgravity cryogenic pipe chilldown data (see ref. ^23^). Heat transfer data^23^ were obtained from flowing LN_2_ through a bare surface stainless steel tube. Recently the UF group has also reported an advance on terrestrial liquid nitrogen transfer line chilldown using low-thermal conductivity Teflon-coated tubes^28,29^ where they revealed a chilldown efficiency improvement up to 109% and savings on liquid nitrogen mass up to 53%. The UF group then added flow pulsing in their liquid nitrogen chilldown experiment^29^ and found that the pulsing can improve the chilldown efficiency up to 66% and also can save the liquid nitrogen mass up to 38%.

**Fig. 1:**
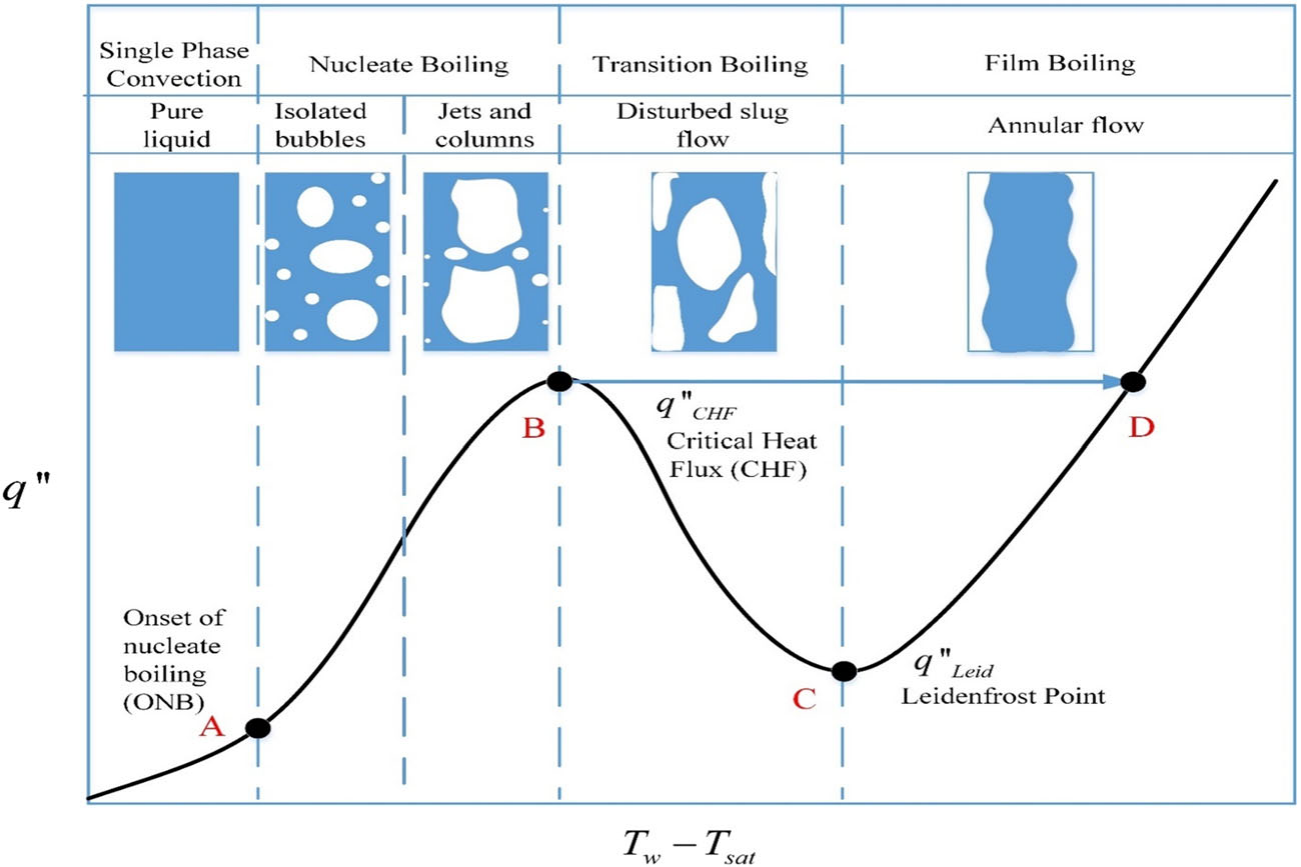
A typical boiling curve and quenching heat transfer characteristics. A quenching (chilldown) process is a liquid-to-vapor phase change phenomenon that is governed by the “boiling curve”. This curve^24^ shows the heat transfer surface heat flux, *q*″, plotted against the surface degree of superheating, *T*_w_ − *T*_sat_, where *T*_w_ is the tube wall inner surface temperature and *T*_sat_ is the saturation temperature corresponding to the boiling fluid bulk pressure. In boiling, if the heating source is externally supplied to the heater surface such as an embedded electrical resistance heating element, the process is heat-flux controlled and follows the route of A → B → D. In contrast, during quenching, the warm wall where the heat comes out does not have a heat supply, therefore, the heat transferring out of the wall can only come internally from the thermal capacity (stored energy) of the wall. The only way to remove heat from the wall is by lowering the inner wall surface temperature using a cooling flow. So quenching is a wall surface temperature-controlled process. Thus, a quenching process follows the route D → C → B → A. During quenching, film boiling is always the first mode of heat transfer encountered due to a relatively very hot surface. Owing to its very low heat fluxes at high wall temperatures^25–27^, film boiling usually dominates the quenching time and cannot be avoided in a traditional chilldown process. As a result, in traditional quenching processes, the thermal energy efficiency is extremely low. According to Shaeffer et al.^5^, the average quenching efficiency that is defined as the ratio of the amount of thermal energy removed from the wall versus the required cooling capability of the cryogen spent in a quenching process is about 8% that highlights the tremendous need to improve the quenching efficiency for many applications that require cryogens as the working fluid.

The current paper is a continued effort following the microgravity experiment of traditional chilldown by Darr et al.^23^. To circumvent the low efficiency of the traditional chilldown process reported by Shaeffer et al.^5^ and further explained in Fig. 1, here we introduce an innovative breakthrough technology that drastically reduces the period of poor heat transfer film boiling by coating the tube inner surface with low-thermal conductivity microscale thin films that expedites the approach of the wall inner surface temperature to the Leidenfrost point, and then to the much more efficient, high heat transfer transition and nucleate boiling period. The theoretical basis of enhancement technique is given in the section of “Methods”.

## Results

### Chilldown curve and boiling curve

**Fig. 2:**
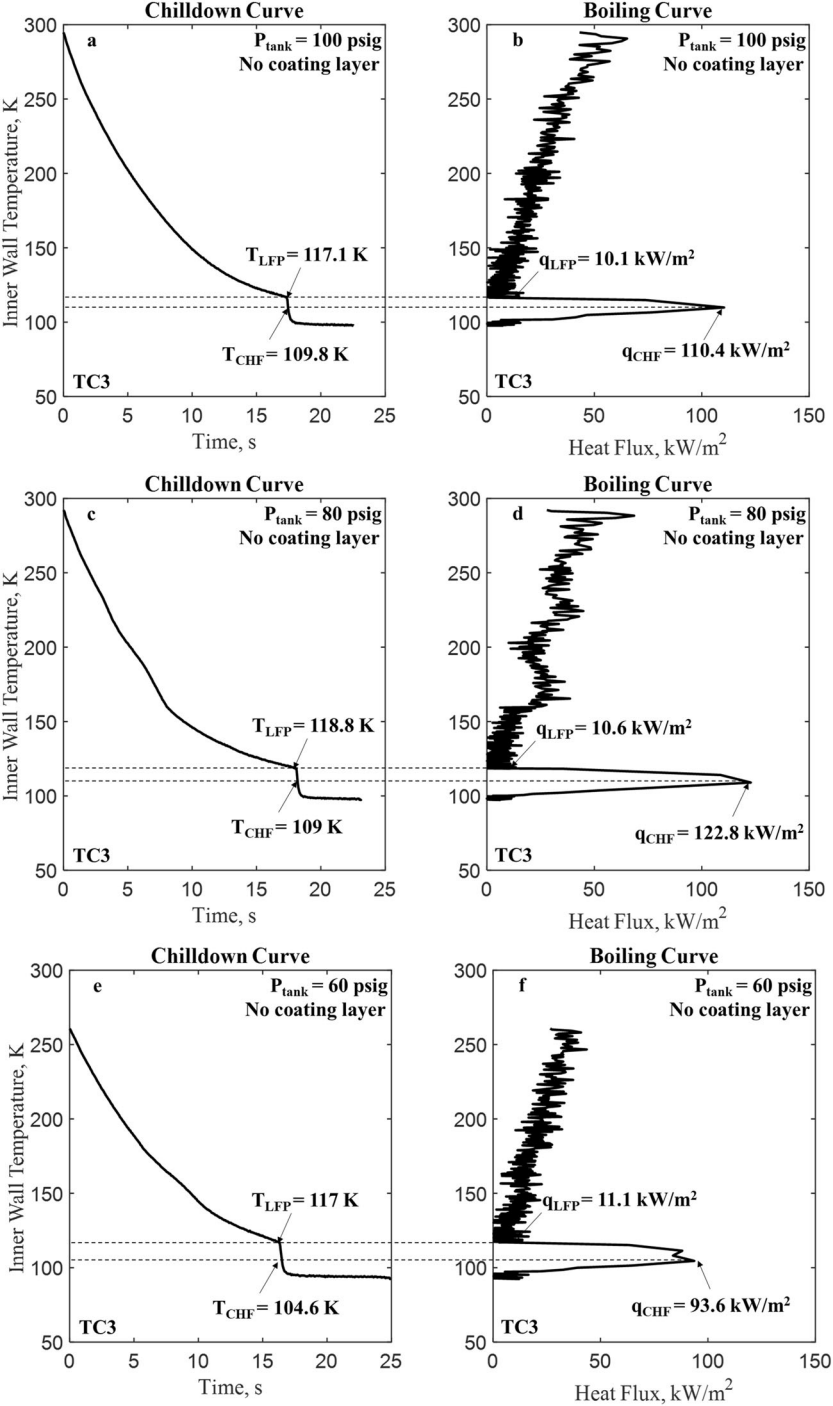
Chilldown curve and boiling curve. A chilldown curve, in general, is a plot of the tube wall temperature change as a function of time or simply it is the tube wall temperature history. In particular, a chilldown curve can be plotted using either the outer or inner tube wall surface temperature history at a certain circumferential location (top, side, or bottom). **a** A typical chilldown curve that records the inner tube wall surface temperature history at TC3 (tube bottom location at the upstream TC station) for the reference base case (Case 1 with bare surface test tube listed in Table 2). For any tube cross-section at a given downstream location, the outer surface is the warmest. As a result, the chilldown time is obtained using the chilldown curve that is based on the tube outer surface temperature. **b** This is the boiling curve for the chilldown curve in Fig. 2a. This boiling curve shares the same tube wall inner surface temperature with its chilldown curve. **c** Chilldown curve for Case 2. **d** Boiling curve for Case 2. **e** Chilldown curve for Case 3. **f** Boiling curve for Case 3.

Before we present and discuss the experimental results, the physics and characteristics of the microgravity quenching process must be delineated first. The performance of a chilldown process is measured by the so-called “chilldown curve” explained in Fig. 2a. For every chilldown curve, there exists a unique corresponding “boiling curve” shown in Fig. 2b. A universal boiling curve has been introduced above in Fig. 1. Both curves are coupled together through a common coordinate of tube inner wall temperature. Please note that LFP (Leidenfrost point, Point C in Fig. 1) and CHF (critical heat flux, Point B in Fig. 1) are marked on both Fig. 2a, b. Both Fig. 2a, b were plotted from data of Case 1 listed in Table 1. Additional chilldown and boiling curves are given in Fig. 2c, d (Case 2) and e and f (Case 3). All the respective LFP and CHF heat fluxes and corresponding temperatures are also indicated in the figures.

Using both chilldown and boiling curves, we can describe the physics of the chilldown process. At the beginning, the film boiling process is the mode of heat transfer that lasted until the Leidenfrost point, followed by the transition boiling that ended at the critical heat flux point. The nucleate boiling then took over after the critical heat flux point. Based on Fig. 2a, the majority of the quenching time is spent in the film boiling regime. After the rewetting (Leidenfrost) point, the heat transfer rates were observed to be much higher in the transition and nucleate boiling regimes that resulted in the fast cooling of the tube wall and a corresponding sudden increase in the slope of the chilldown curve at LFP. As can be seen in Fig. 2a, the Leidenfrost point is located just ahead of the sharp turn of the chilldown curve, and the almost vertical line belongs to the transition and nucleate boiling regimes with the CHF separating the two regimes. From Fig. 2a, the film boiling regime lasted 17.3 s and the transition and nucleate boiling together lasted less than 1 s. The results presented below demonstrates the ability of the thin-film coating to shorten the film boiling period.

### Quality of microgravity environment during parabolic flights

**Fig. 3:**
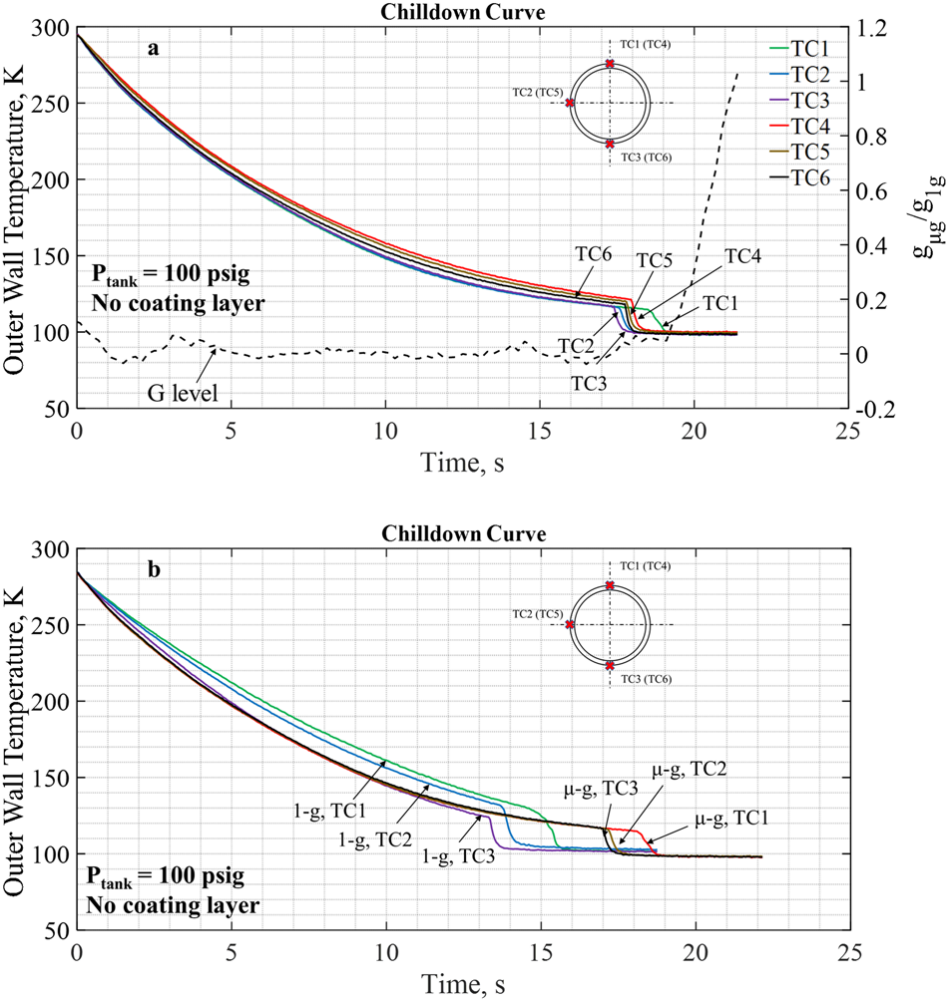
Quality of microgravity environment in parabolic flights. **a** Three chilldown curves are plotted using the temperature data registered by the three thermal couples depicted in Fig. 7d where TCs 1 (top), 2 (side), and 3 (bottom) are all located at the same axial location (upstream TC station). Three additional chilldown curves from the three TCs 4 (top), 5 (side), and 6 (bottom) placed at the downstream location are also plotted for comparison. The chilldown curves provide the tube wall outer surface temperature histories during chilldown. The *z*-acceleration (upward in the aircraft coordinate system) is also plotted versus time in this figure. The acceleration levels or g-jitters of the microgravity period are within ±0.01*g* and all other flights had the same acceleration levels. **b** The three microgravity chilldown curves from Fig. 3a are plotted together with their terrestrial (1−*g*) counterparts (horizontal tube test chilldown curves^28^).

The high quality of the microgravity environment provided by the Zero-g Corporation’s Boeing 727 aircraft performing parabolic flights is demonstrated in Fig. 3a, b where chilldown curves for Case 1 (Supply tank pressure, *P*_in_ = 100 psig and bare surface tube with no coating) are shown. It should be pointed out first that the tube test section was mounted parallel to the plane floor so that the acceleration vector was always perpendicular to the flow direction. Any level of gravitational acceleration would result in a stratified two-phase flow pattern where the liquid settles down to the bottom of the tube and vapor buoys to the top. In Fig. 3a, a set of three microgravity chilldown curves are plotted showing the local temperature histories registered by the thermocouples located on the top (TC1), side (TC2), and bottom (TC3) tube surfaces at the same upstream TC station during chilldown in microgravity. In general, all three chilldown curves shown in Fig. 3a collapsed virtually into one single curve from the beginning up to 17.5 s when the microgravity period ended. During the microgravity period, the heat transfer process is in the film boiling regime (between C and D in Fig. 1) and the flow pattern belongs to the so-called “inverted annular flow” where the vapor travels on the outer annulus of the flow blanketing the tube inner surface and the liquid fills the flow central core portion. The symmetric inverted annular flow is a strong indication that the two-phase flow is relatively stratification-free and, therefore, virtually gravity independent. When the microgravity period ended, the aircraft transitioned into a high 1.8*g* condition (see Fig. 7e) that results in a stratified two-phase flow pattern where the liquid settles down to the bottom of the tube. The stratified flow enables the rewetting of bottom surface (TC3) first by liquids that results in the chilldown time to be the shortest for the bottom location, the side location (TC2) to be the next, and the top (TC1) relatively the longest which is exactly the results found in Fig. 3a. Figure 3b provides another proof where the three microgravity curves are plotted together with their three terrestrial counterparts^28^. For the terrestrial horizontal tube test^28^, the three chilldown curves are separated from one another from the beginning in the film boiling regime with the top wall (TC1) showing the slowest rate of chilldown and the bottom wall (TC3) the fastest, which demonstrates the typical stratification effects due to gravity. However, three temperature readings for the microgravity test are bundled closely together resembling a single curve (also shown in Fig. 3a) which shows that the buoyancy force was negligible, similar to microgravity space conditions.

Quantitatively, the measured *z*-acceleration (upward in the aircraft coordinate system) given in Fig. 3a is within ±0.01*g*. Based on the converged chilldown curves and measured acceleration levels, we are confident that the environment facilitated by the parabolic flight closely resembled that of space microgravity.

Three additional chilldown curves from the three TCs 4 (top), 5 (side), and 6 (bottom) placed at the downstream location are also plotted in Fig. 3a for comparison with the upstream results. It can be seen that the downstream chilldown curves show more gravity effects as the three curves do split slightly into three separate curves after 8 s. The reason for the separation among the three curves is that primarily in a convective flow, the stratification effect due to residual gravity (0.01*g*) is accumulative with the residence time of flow in the tube as the two-phase flow spends more time to reach the downstream location.

### Breakthrough impacts on chilldown efficiency by coatings

**Fig. 4:**
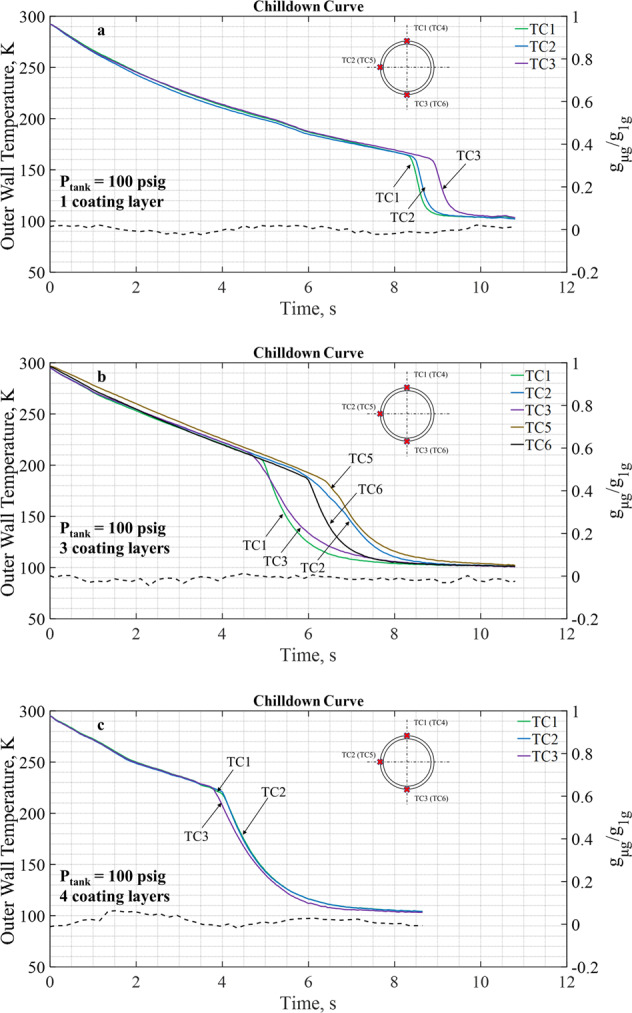
Chilldown curves for test tubes with different thicknesses of coating layers. On each figure there are three chilldown curves generated with data collected from the three TCs (located at top, side, and bottom) with all at the upstream TC station under the same inlet pressure of 100 psig. **a** Test tube with one coating layer, **b** test tube with three coating layers, and **c** test tube with four coating layers. The chilldown curve of the bare tube surface case is given in Fig. 3a.

First, we examine the impact for different coating thicknesses. Figure 4 presents the chilldown curves for three coated tubes under the same tank pressure of 100 psiag in microgravity. Due to the coatings, all chilldown processes shown in Fig. 4 for a tank pressure of 100 psig were completed in less than 17.5 s that is the period of microgravity during parabolic flights. Thus, all chilldown processes proceeded completely under microgravity. As explained for Fig. 3a and can be seen here in Fig. 4 during the initial film boiling period, the tube wall outer surface temperature histories at TC1, TC2, and TC3 are almost merged into a single curve due to the axisymmetric inverted annular flow pattern in microgravity. By comparison, we can clearly see that the coatings shortened the film boiling time periods and raised the outer wall temperatures at which the Leidenfrost points were reached. As shown in Figs 3a and 4a–c, we found that the Leidenfrost points were reached for bare surface, 1-, 3-, and 4-Layer test tubes at 17, 8.5, 5, and 4.1 s, respectively, after the chilldown started and the corresponding outer wall surface temperatures were 120, 160, 205, and 220 K, accordingly. In summary, the coatings expedited the chilldown processes and we found that the thicker the coating the faster the chilldown reached completion. The benefit of the coatings appeared to have diminishing returns as the coating layers got thicker.

Also shown in Fig. 4b are additional chilldown curves for the downstream location. Only chilldown curves from TC5 and TC6 are given as TC4 malfunctioned during the experiment. Similar to comparison discussed for Fig. 3a, the downstream chilldown curves do show more separations due to accumulative stratification effects.

**Fig. 5:**
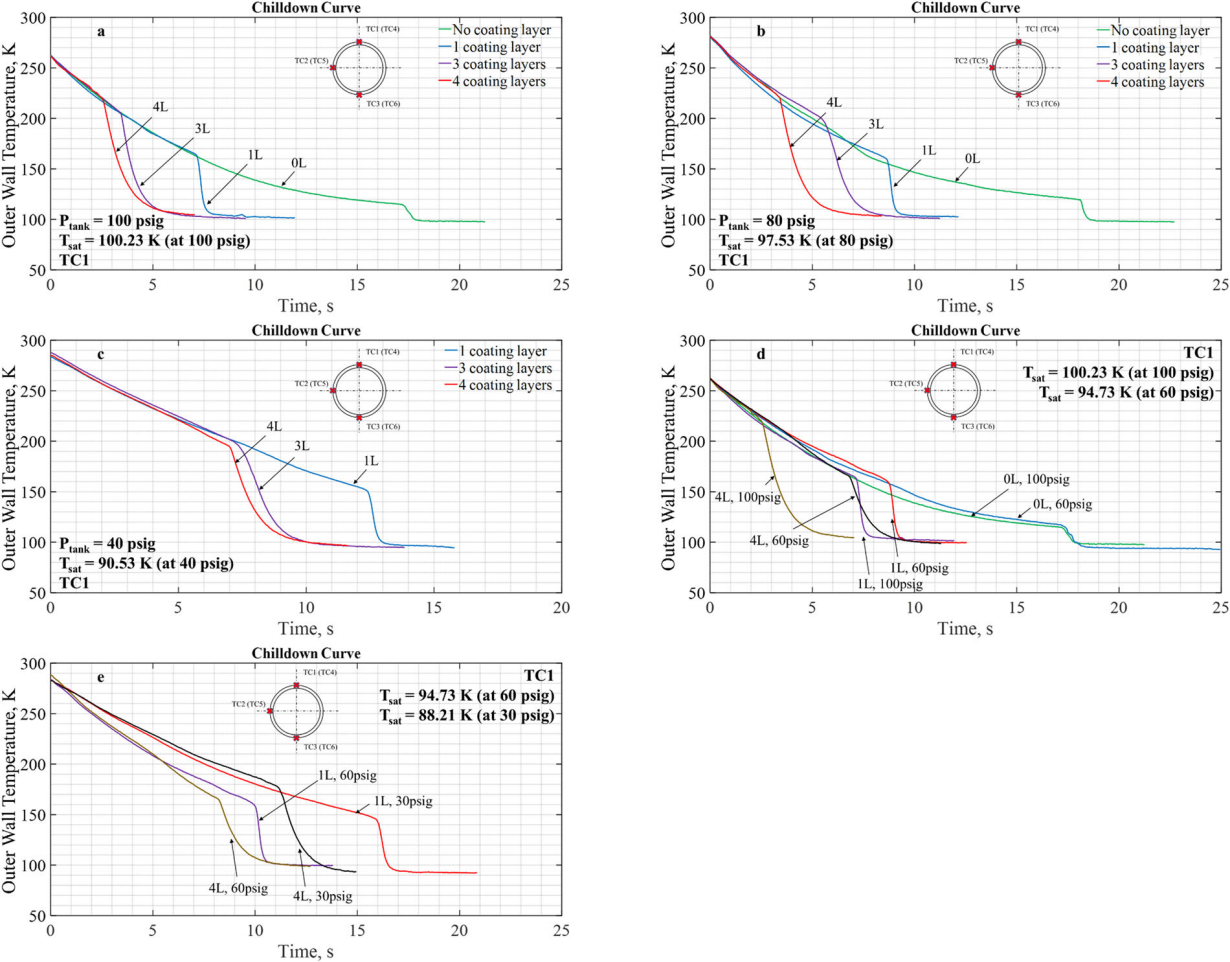
The chilldown curves for various coating layer thicknesses and different source pressures. Chilldown results for inlet pressures of 100, 80, and 40 psig are shown in **a**, **b**, and **c**, respectively. For **a** and **b**, each presents four chilldown curves that correspond to three coating thicknesses plus one bare surface without any coating, while **c** only presents three curves as mentioned in the “Methods” section that the bare surface case was not performed for the 40 psig case due to lack of liquid nitrogen supply. Panels **d** and **e** plot the results for TC1 at the upstream station where chilldown curves are shown for 0, 1, and 4 tubes under 100, 60, and 30 psig. **a** 100 psig inlet pressure. **b** 80 psig inlet pressure. **c** 40 psig inlet pressure. **d** 100 psig and 60 psig inlet pressures. **e** 60 psig and 30 psig inlet pressures.

Next, we look into the coating impact under various source pressures. Different source pressures resulted in different coolant mass flow rates. The higher the inlet pressure, the high the mass flow rate. Based on Fig. 5a–c, the list below summarizes the effects of different coating thicknesses under a given source pressure.

1. It is clear that at a given inlet pressure the reduction in chilldown time is relatively proportional to the total thickness of the coating layers. Therefore, the thicker the coating is, the shorter the chilldown time results.
2. The basic single trend for all three inlet pressures is that the chilldown rates during the film boiling period are all very close to one another, the only difference that sets them apart is that the thicker the coating, the quicker the tube inner surface reached the LFP, thus correspondingly the LFP is at a higher outer surface temperature.
3. It is also observed that the scenario of diminishing return can be applied here when increasing the thickness of the coatings. The largest reduction in chilldown time is realized when going from a bare surface to a 1L coating and then the chilldown time reduction gets smaller as more layers are added.

Furthermore, the effectiveness of the tube coating is evaluated for various inlet pressures. Based on Fig. 5d, e, for a given coating layer thickness, the general finding on the effectiveness of the coating for different inlet pressures is listed below.

1. With the inlet pressures set at 100, 60, and 30 psig, a consistent trend suggests that for a given coating layer thickness, the higher the inlet pressure, the shorter the chilldown time was measured. This observation is basically due the fact that higher inlet pressures would induce higher mass flow rates that results in higher convective cooling heat transfer rates.
2. However, with the bare surface tube case, we found that the chilldown time was not a sensitive function of the different inlet pressures.
3. Similar results were found also for inlet pressures of 80 and 40 psig.

### Advancement of chilldown thermal energy efficiency by coatings

The thermal efficiency of a cryogenic quenching system is completely determined by the cooling heat transfer rates between the tube wall and the liquid nitrogen flows. As a result, we measure the chilldown system performance by its thermal energy efficiency, *η*_CD_, that represents the percent of the available coolant total chilldown capacity that is actually used in removing the heat from the tube wall. More specifically, the chilldown thermal energy efficiency in %, *η*_CD_, is therefore defined as

1 $$\eta _{\mathrm {CD}} = \frac{{Q_{{\mathrm{removed}}}}}{{Q_{\mathrm {available}}}} \times 100\%$$

In the above, *Q*_removed_ is the entire thermal energy (heat) removed from the total system mass that has to be chilled down during the entire chilldown period and is defined as

2 $$Q_{{\mathrm{removed}}} = \left( {M_{\mathrm {tube}} + {\mathrm{0}}{\mathrm{.3}}M_{\mathrm {valve}}} \right)c_{\mathrm {p},\,\mathrm {SS}}\left( {T_{{\mathrm {initial}} - T_{\mathrm {final}}}} \right)$$

**Fig. 6:**
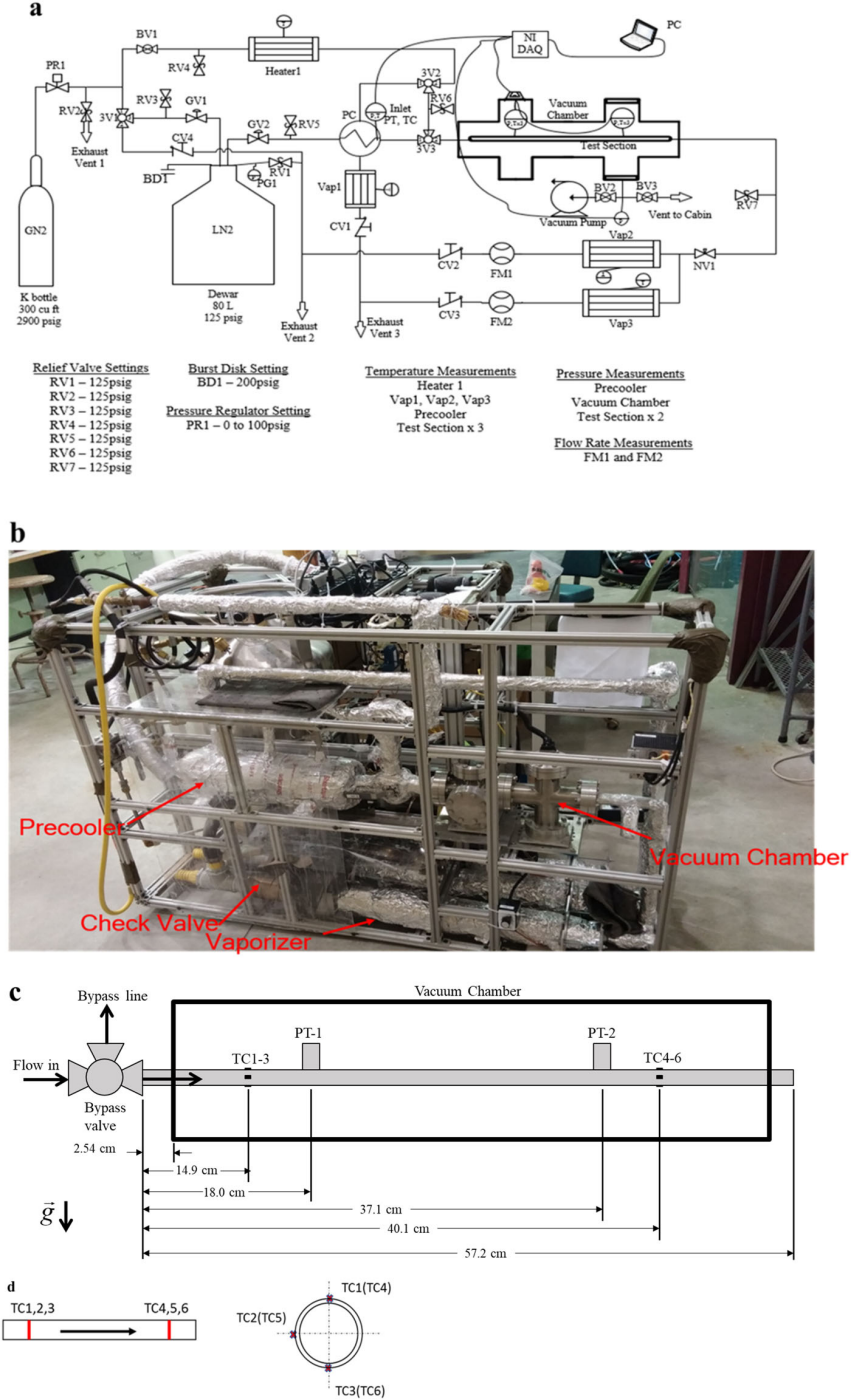
Experimental system. **a** Fluid system schematic. The valves and important components of the fluid network. Relief valve settings, the burst disk setting, and pressure regulator settings are also included. BD burst disk, BV ball valve, CV check valve, DAQ data acquisition unit, FM flow meter, GN2 gaseous nitrogen, GV globe valve, LN2 liquid nitrogen, NV needle valve, PC pre-cooler, PG pressure gauge, PR pressure regulator, PT pressure transducer, RV relief valve, TC thermocouple, Vap vaporizer, 3V three-way valve. **b** Photograph of experimental system. **c** Test section dimensions and details. **d** Schematic showing the placement of six TCs. There are two TC stations, one upstream and one downstream. Three TCs are placed at each TC station in the form of top, side, and bottom separations.

In the above, (*M*_tube_ + 0.3*M*_valve_) is the total system chilldown mass, where *M*_tube_ is the mass of the test tube section between the three-way globe valve and the upstream TC station, which has a length of 14.9 cm (see Fig. 6c) and *M*_valve_ is the mass of the three-way globe valve (shown in Fig. 6c), respectively. For the globe valve, a factor of 0.3 is applied to the valve mass, as we estimated that only 30% of the valve mass needed to be chilled down when the chilldown process began, as part of the valve is in contact with the LN_2_ before opening the valve to start the experiment. Therefore, we believe that globe valve is partially (about 70%) chilled down during precooling and reheating of the test section where the liquid nitrogen filled the path all the way to the valve. *c*_p_,_SS_ is the specific heat of stainless steel for both the tube and valve materials. *T*_initial_ and *T*_final_ are the initial and the final temperatures of the system during the entire chilldown period, respectively. It is noted that the end of chilldown temperature, *T*_final_, is the saturation temperature of liquid nitrogen corresponding to the local pressure. *Q*_available_ is the total available quenching capacity supplied by the coolant during the chilldown process. It is defined as

3 $$Q_{\mathrm {available}} = M_{\mathrm {coolant}}h_{\mathrm {fg}}$$

where *M*_coolant_ is the total mass of coolant supplied or consumed during the entire chilldown period and it is defined below.

4 $$M_{\mathrm {coolant}} = {\int}_0^{t_{\mathrm {End}}} {\dot m(t){\mathrm d}t}$$

where $\dot m(t)$ is the measured time-dependent coolant mass flow rate during the chilldown period and *t*_End_ is the time at the end of chilldown. Therefore, *M*_coolant_ is the total mass of coolant consumed during the entire chilldown process. Since *h*_fg_ is the latent heat of vaporization per unit mass, that means *Q*_available_ is the total available quenching capacity of the liquid nitrogen coolant of mass, *M*_coolant_, supplied during the chilldown process.

The chilldown efficiencies for various source tank pressures and different coating thicknesses (bare surface, 1-, 3-, and 4-Layer) are listed in Table 1. It is important to report that the range of the relative uncertainty (%) of the chilldown efficiency is estimated between ±14.71 and ±15.29%. First, for the bare surface tube case, we note that the chilldown efficiencies do not seem to be affected to any significant extent by the variation of the source pressures as the efficiencies fluctuated slightly around 25.7% for the three inlet pressures. The same trend was found for the terrestrial counterpart experimental results^28^. Since we did not perform the 40 and 30 psig experiments for the bare surface tube, we have listed 25.72% with an (*) as the estimated chilldown efficiencies for both 40 and 30 psig tank pressure cases. For the effects of the coating layers, the efficiency increases with increasing number of layers for bare surface, 1- and 3-Layer cases. However, between 3- and 4-Layer, enhancement saturation seems to have been reached. However, the efficiencies for the coated tubes also do not seem to have a clear dependence on the inlet pressure as the uncertainty is about ±15%. In general, the efficiencies cover a range between 24.38 and 70.87%. If we were to use the averaged efficiency over the five inlet pressures, the averaged efficiencies are 25.72%, 40.33%, 54.36%, and 50.89% for bare surface, 1-, 3-, and 4-Layer tubes, respectively. More specifically, let us define the percent increase in efficiency for the coated tube over the non-coated bare surface tube case under the same inlet pressure as follows and the results are also given in Table 1.

5 $${\mathrm{percentage}}\,{\mathrm{increase}}\,{\mathrm{in}}\,{\mathrm{efficiency}} = \frac{{\eta _{x{\mathrm L}} - \eta _{0{\mathrm L}}}}{{\eta _{0{\mathrm L}}}} \times 100\%$$

where *η*_*x*L_ and *η*_0L_ are the thermal efficiencies for the *x*-Layer-coated tube and the bare surface tube, respectively. For the percentage increase in the efficiency, the minimum is 33.15% for 1-Layer and 60 psig inlet pressure and the maximum is 175.54*% for 4-Layer and 40 psig inlet pressure. It is noted that the results listed in Table 1 include those that are based on estimated bare tube efficiencies so they are indicated by an (*). For the averaged percent increase in efficiency over the five inlet pressures, they are 56.77%, 111.43%, and 97.93% for 1-, 3-, and 4-Layer, respectively.

**Table 1:** The chilldown efficiencies, *η*_CD_; the percentage increase in *η*_CD_ over the base case, 0L; total LN_2_ consumed for chilldown process, *M*_coolant_; and percent reduction in LN_2_ consumption over the base case, 0L, for different coating layers and source pressure.

*P*_in_ psig	100	80	60	40	30	Average
**0** **L (no coating)**						
*η*_CD_ (%)	25.57	24.38	27.21	25.72*	25.72*	25.72
*M*_c__oolant_ (kg)	1.1269	1.1497	0.8938	1.07*	1.07*	1.0621
**1** **L coating**						
*η*_CD_ (%)	NA	35.90	36.23	42.77	46.42	40.33
Percentage increase in *η*_CD_ (%)	NA	47.25	33.15	66.29*	80.40*	56.77
*M*_coolant_ (kg)	NA	0.7673	0.7419	0.6146	0.5642	0.672
Percentage reduction in *M*_coolant_ (%)	NA	33.26	16.99	42.6*	47.3*	35.04
**3L coating**						
*η*_CD_ (%)	55.17	45.07	48.88	62.89	59.77	54.36
Percentage increase in *η*_CD_ (%)	115.76	84.86	79.64	144.52*	132.39*	111.43
*M*_coolant_ (kg)	0.5206	0.6147	0.5525	0.4192	0.4376	0.5089
Percentage reduction in *M*_coolant_ (%)	53.80	46.53	38.19	60.8*	59.1*	51.68
**4L coating**						
*η*_CD_ (%)	45.14	42.20	45.75	70.87	50.50	50.89
Percentage increase in *η*_CD_ (%)	76.54	73.09	68.14	175.54*	96.35*	97.93
*M*_coolant_ (kg)	0.6316	0.6555	0.5964	0.3714	0.5185	0.5547
Percentage reduction in *M*_coolant_ (%)	43.95	42.99	33.27	65.2*	51.5*	47.38

### Significant reductions in propellant consumption by the coatings

For thermal energy management of the cryogenic propellants in space, the savings in propellant mass consumptions when the supply onboard is limited are of the highest concerns. Although the total cryogen mass consumption is directly proportional to the chilldown thermal efficiency, it is meaningful to present the results of required cryogen mass consumption that would provide the spacecraft thermal-fluid system design engineers with the most important design criterion in cryogen conservation and efficient use. Next, we present quantitatively the cryogen mass consumption together with mass reduction in cryogen consumption due to thin-film coatings.

As mentioned above, the total amount of coolant consumed during chilldown is *M*_coolant_ and the percent reduction in coolant consumption for *x*-Layer coating over the bare surface tube reference case under the same inlet pressure is defined below.

6 $${\mathrm{percentage}}\,{\mathrm{reduction}}\,{\mathrm{in}}\,{\mathrm{mass}}\,{\mathrm{for}}\,{x{\mathrm{L} = }}\frac{{M_{\mathrm {coolant},\,{\mathrm {bare}}\,{\mathrm {surface}}} - M_{{\mathrm {coolant}},\,x - {\mathrm {Layer}}}}}{{M_{{\mathrm {coolant}},\,x - {\mathrm {Layer}}}}} \times 100\%$$

Table 1 again lists both the total liquid nitrogen consumed and percentage reduction in liquid nitrogen consumption, respectively. We need to mention that the range of the relative uncertainty (%) of the measured mass flow rates is estimated between ±2.57% to ±4.91%. For the total liquid nitrogen consumed, the trends are more clear and similar to those of the chilldown efficiency. The required liquid nitrogen total amount decreases with decreasing inlet pressure and also decreases with increasing number of coating layers. Again, there is no clear distinction between 3- and 4-Layer cases. It is more meaningful to look at the percentage reduction in the cryogen mass used, the trends are similar to those of the total liquid nitrogen consumed. It is found that the minimum is 16.99% for 1-Layer and 60 psig inlet pressure and the maximum is 65.2% for 4-Layer and 40 psig inlet pressure. For the averaged percent reduction in cryogen mass consumption over the five inlet pressures, they are 35.04%, 51.68%, and 47.38% for 1-, 3-, and 4-Layer, respectively. As expected these results follow the same trends as those discussed above for the chilldown efficiencies.

## Discussion

After analyzing the obtained experimental data, we have found a general trend that the quenching heat transfer enhancement that was reflected in the increase of the chilldown efficiency is a function of the coating layer thickness, that means the thicker the coating layer the higher the chilldown efficiency. But it is important to note that there seems to be a diminishing return tendency that the rate of enhancement dropped off as the coating layer got thicker. Based on the physics, there are two opposing heat transfer effects that are involved in the quenching cooling of a tube with a composite two-layer structure. The first effect is the thermal insulating property of the low-thermal conductivity thin layer that induces a large and quick drop of the tube inner surface temperature at the beginning of chilldown by limiting the heat flow from the bulk of the tube wall to the tube inner surface. We know that the thicker the coating layer the faster the inner surface temperature drops. This lower inner surface temperature enables the surface to quickly reach the Leidenfrost point and also the switch from the film boiling regime to transition boiling and then to nucleate boiling regimes. The second effect is the conduction heat transfer from the bulk of the tube wall to the coolant through the tube inner surface that actually decides the overall cooling rate. The heat conduction through the two composite layers is inversely proportional to the coating thickness that requires the Teflon coating to be as thin as possible. Based on the above discussion, there should exist an optimal thickness for the low-thermal conductivity coating that balances these two opposing effects such that the coating is just thick enough to quickly drop the tube inner surface temperature to the Leidenfrost point, but it is still relatively thin not to significantly decrease the heat flow from the bulk of tube wall to the coolant after the initial period. For the current coated tube design, we suggest that the optimum coating thickness falls between those of 3- and 4-Layer coatings.

Another important finding is that the chilldown efficiency and the corresponding reduction in cryogen mass consumption increase with decreasing inlet pressure and cryogen mass flow rate.

We have demonstrated the feasibility of an innovative concept for the advance of an efficient thermal energy management process for cryogenic rocket propellants in a simulated space microgravity environment. The low-thermal conductivity Teflon microfilm coating on the inner surface of a cryogenic transfer pipe was shown to be able to achieve the maximum average quenching thermal energy efficiency of 54.36% that is 111.43% improvement over the traditional, existing quenching approach using a bare surface tube with an average efficiency at 25.72%

From a more practical perspective on rocket propellant thermal management in space, the 111.43% improvement in efficiency translates into a maximum propellant average mass consumption saving of 51.68% over the existing technique using a bare surface pipe that is a leapfrogging advance towards the goal of conserving precious propellant consumption for enabling long-range missions to mars and beyond.

## Methods

### Theoretical basis of methods

A physical model based on the transient one-dimensional heat conduction in a thin solid slab^30^ was developed to explain the theoretical basis of incorporating a low-thermal conductivity microscale thin-film surface coating. The readers are referred to Chung et al.^29^ for the details of the model. The model predicted that the magnitude of the tube inner surface temperature drop is more than 15 times larger than that of the bare surface tube during the same initial period.

Based on the model prediction, we can take advantage of the thermal insulating effect of the low-thermal conductivity thin layer of Teflon coating to facilitate a quick large drop of the tube inner surface temperature at the beginning of the quenching by restricting the heat flow from the bulk of the tube wall to the tube inner surface. As a result, the inner surface temperature of a coated tube would reach the Leidenfrost point much quicker than does the bare surface tube with no coating. This much lower inner surface temperature enables the quenching process to move quickly from the poor heat transfer film boiling regime to the Leidenfrost point, and then to the much higher heat transfer transition boiling and nucleate boiling regimes. Therefore, the poor heat transfer film boiling period is shorten substantially for a coated tube to allow the much more efficient transition and nucleate boiling to take over to complete the quenching process.

### Experimental apparatus and procedure

The current parabolic flight experimental system was built with minor modifications and improvement to our original rig used by Darr et al.^13,14^ and Darr et al.^23^ for similar cryogenic chilldown experiments with bare surface test tubes only in terrestrial gravity and microgravity, respectively. A flow network and instrumentation component schematic of the experiment system is shown in Fig. 6a. To avoid duplication, the readers are referred to Darr et al.^23^ for a detailed description of the flow network, and the function and operation of each component. Specifically, it is important to note that a subcooler was placed before the entrance of the test section. The function of the subcooler was to insulate the tubing upstream of the test section from ambient parasitic heat input and to make sure that the fluid entering the test section was vapor free, subcooled liquid that provides a precise inlet boundary condition for the test tube during chilldown. A photograph of the experimental system used in the parabolic flights is given in Fig. 6b.

As shown in Fig. 6c, d, the test section was a 57.2 cm long, 1.270 cm OD, 1.168 cm ID 304SS tube. A length of 2.54 cm of the test section tube protruded out of each side of the vacuum chamber. Six thermal couples (TCs) were soldered to the outside of the test tube, with three placed at an axial distance of 14.9 cm from the test section inlet (upstream TC station), and the other three placed at 40.1 cm from the inlet (downstream TC station). As detailed in Fig. 6d, for each station, the TCs were spaced out radially in 90° increments such that each station had a top, side, and bottom TC. Two cryogenic rated pressure transducers (PTs) were connected to two short pieces of 304SS tube protruding perpendicularly from the test section, one near each TC station. These tubes were welded to the test section.

As to the 316SS vacuum chamber that housed the test section, the purpose of the vacuum chamber was to reduce parasitic heat leak that would reduce the uncertainty in the calculation of chilldown heat flux. A diaphragm pump and molecular turbopump were used to bring the air pressure inside the chamber down to approximately 1 Pa. This near vacuum pressure reduced the parasitic heat leak due to conduction between the test section and the air inside the vacuum chamber to less than 10% of the lowest measured convective heat flux, which occurred during film boiling at the lowest flow rate that was tested. Parasitic heat leak was less than 1% of the measured value for most of the data points.

**Fig. 7:**
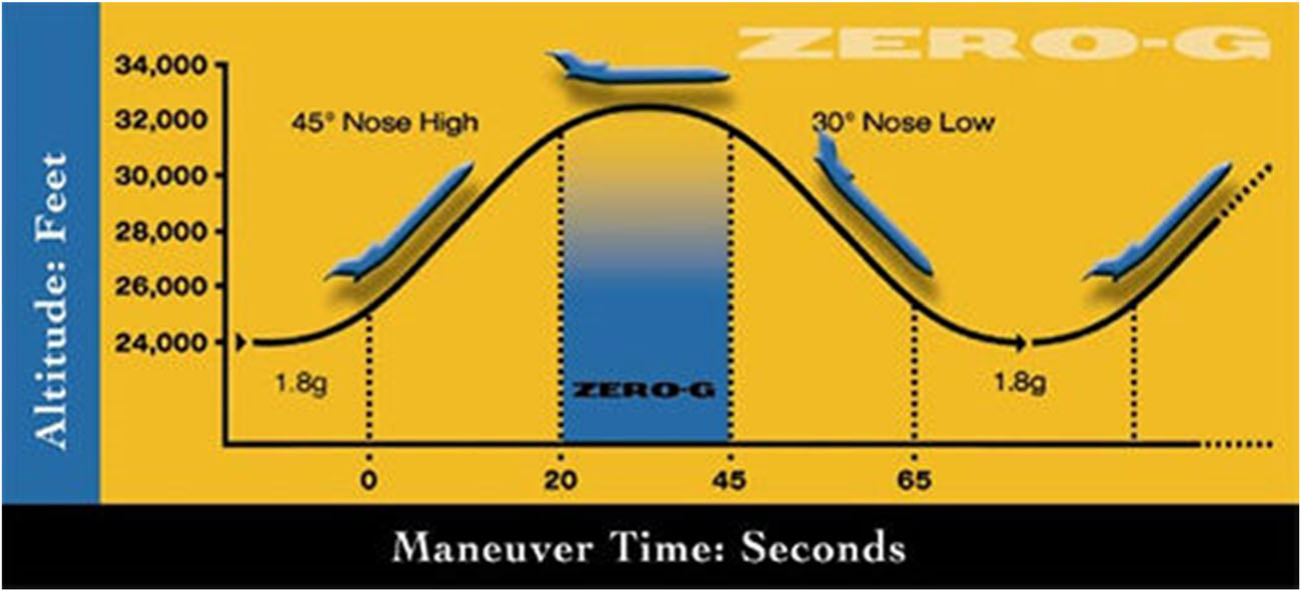
Schematic of parabolic flight variable gravity levels versus time. The 25-s micro-g period is sandwiched by two high 1.8*g* periods.

Figure 7 provides the information on the parabolic flight gravity-level versus time characteristics^31^. The microgravity window is nominally about 25 s (it is about 18 s in our flights for lower gravitational acceleration) and it is sandwiched by two high 1.8*g* periods. Except for running the experiments in synchronization with microgravity windows shown in Fig. 7 for parabolic flights, the rest of the experimental procedure is identical to that followed in our previous chilldown experiment. So, the readers are again referred to Darr et al.^23^ for the details of the experimental procedure.

#### Microscale thin-film coating of tube inner surface

For the current experiment, the original bare surface stainless steel test tube was additionally coated with low-thermal conductivity microscale thin-film Teflon layers on the tube inner surface. Specifically, the coating material was made of fluorinated ethylene propylene by DuPont and classified by DuPont as Teflon 959G-203 that is a black color paint and has a thermal conductivity of 0.195 W/mK (DuPont publication^32^).

**Fig. 8:**
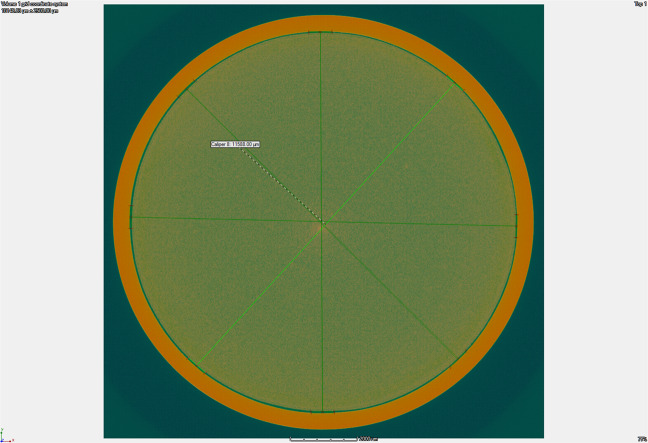
A typical tube cross-section image. X-ray computer tomography image of a tube cross-section.

We have made coated tubes with four different coating layer thicknesses that are identified as one-layer (1-Layer), two-layer (2-Layer), three-layer (3-Layer), and four-layer (4-Layer) coatings. For example, the 4-Layer coating went through the pour and drain coating process four separate times. The thicknesses of the coating layers were measured by X-ray computer tomography. A typical cross-sectional image is given in Fig. 8. The average coating thicknesses and respective uncertainties for 1-, 2-, 3-, and 4-Layer tubes were measured at 1533 ± 0.6, 25.8 ± 0.7, 45.28 ± 0.7, and 64.8 ± 0.7 µm, respectively.

#### Microgravity flight experiments completed

The University of Florida flight team led by Professor Jacob Chung performed parabolic flight experiments in four flights during the week of 20–24 March 2017. Since we only had four flights, four different test tubes were used. Among them, three were made with different inner surface modifications (one-layer coating (1-Layer), three-layer coating (3-Layer), and four-layer coating (4-Layer)) and the fourth one was the bare surface tube without any coating to serve as the baseline case for evaluating the coating effects. The reason for not using the 2-Layer tube is that based on our own terrestrial counterpart experimental results^28,29^, we found that the chilldown efficiency versus coating thickness curve is a relatively smooth, monotonically rising one that shows the efficiency increases with increasing coating thickness, but the rate of increase (the slope of the curve) decreases with coating thickness that indicates the effect of coating has become saturated (a case of diminishing return) as the coating gets thicker. Therefore, in order to develop a complete chilldown efficiency versus coating thickness curve, it is important to maximize the range of coating thickness such that the high end of the coating thickness can reach to the saturation zone. By this way, we can estimate the chilldown efficiency for the 2 L tube with a reasonable accuracy by a safe interpolation scheme. For each coated tube, five different inlet pressures of 100, 80, 60, 40, and 30 psig, respectively, were applied that resulted in five different LN_2_ mass flow rates during chilldown.

Due to a limited amount of liquid nitrogen supply on board and longer chilldown times, only 100, 80, and 60 psig were performed for the bare surface baseline case during the first flight as the chilldown time is relatively longer for the bare tube case such that the LN_2_ supply on board ran out after the 60 psig test. So, a total of 18 successful chilldown tests were run and completed in the four microgravity flights. Table 2 lists the surface coating applied, inlet pressure setting, the time-averaged (over the duration of reduced gravity period) inlet liquid Reynolds number, Re_in_ (computed from Re_in_ = *G*_ave_*D*/*μ*_l_, where *G*_ave_ is the time-averaged mass flux, which is the mass flow rate divided by the flow cross-sectional area of the test section, *D* is the inner diameter of the test section, and *μ*_l_ is the saturated liquid dynamic viscosity based on the inlet pressure), the time-averaged mass flux *G*_ave_, end of chilldown time, *t*_end_, and LN2 inlet degree of subcooling,*T*_in_, for each of the 18 cases.

**Table 2:** Test conditions for the 18 tests, representing low flow rate, medium flow rate, and high flow rate tests.

Case	Coating applied	*P*_in_, psig	Re_in_	*G*_ave_, kg/m^2^ s	*t*_end_, s	*T*_in_, K
1	No coating	100	63,030	455	21.0	95.95
2	No coating	80	58,909	436	21.5	89.82
3	No coating	60	35,073	300	21.55	90.96
4	1L coating	100	NA	NA	10.6	NA
5	1L coating	80	62,901	454	11.5	96.07
6	1L coating	60	44,827	365	13.0	92.43
7	1L coating	40	26,763	249	15.05	88.80
8	1L coating	30	16,061	168	19.05	85.65
9	3L coating	100	58,704	422	9.70	96.26
10	3L coating	80	55,149	413	10.35	94.53
11	3L coating	60	44,326	355	11.45	92.56
12	3L coating	40	25,480	232	13.0	89.24
13	3L coating	30	19,455	195	15.75	86.54
14	4L coating	100	77,968	538	9.2	93.65
15	4L coating	80	63,406	459	9.6	92.19
16	4L coating	60	36,657	302	11.55	90.86
17	4L coating	40	26,792	249	12.0	89.21
18	4L coating	30	17,203	188	15.55	83.86

Conditions are based on the upstream TC station.

### Experimental uncertainties

As mentioned above, the current experimental system is virtually identical to that employed in Darr et al.^13,14^, the readers are referred to Darr et al.^13,14^ for uncertainties on those independent, directly measured quantities, such as temperatures and pressures.

### Tube inner surface temperature and heat flux estimation

Burggraf^33^ demonstrated that for a hollow cylinder that undergoes a temperature change due to heating or cooling at the inside, both the inner wall temperature and the inner wall heat flux at a local axial position can be calculated from knowledge of the corresponding outer wall temperature, using a Taylor series expansion so that these quantities are solved as a function of the outer wall temperature and its time derivatives as well as the thermodynamic properties of the tube material. This method is particularly accurate for the current experiment because only a few terms of the expansion are necessary for thin tubes. However, this method cannot be applied for coated tubes. This method has been used for several of our previous terrestrial and microgravity chilldown experiments^8,11–14,23,27–29^ with success. Readers are referred to those papers for details.

### Reporting summary

Further information on research design is available in the Nature Research Reporting Summary linked to this article.

## Supplementary information

Reporting Summary

## Data Availability

The authors declare that the data supporting the findings of this study are available within the paper.
